# Advance directives from haematology departments: the patient’s freedom of choice and communication with families. A qualitative analysis of 35 written documents

**DOI:** 10.1186/s12904-017-0265-1

**Published:** 2018-01-02

**Authors:** S. Trarieux-Signol, D. Bordessoule, J. Ceccaldi, S. Malak, A. Polomeni, J. B. Fargeas, N. Signol, H. Pauliat, S. Moreau

**Affiliations:** 1 Haematology and cellular therapy Department, CHU Limoges, 2 avenue Martin Luther King, 87000 Limoges, France; 2 Libourne Hospital, 112, rue de la Marne BP 199, 33505 Libourne, Cedex France; 3 Curie Institute, 26 Rue d’Ulm, 75005 Paris, France; 4 Saint Antoine Hospital, 184 Rue du Faubourg Saint-Antoine, 75012 Paris, France; 5 Saint Yrieix la Perche Hospital, Place du Président Magnaud, 87500 Saint Yrieix la Perche, France; 6 Saint-Junien Hospital, 12, rue de Châteaubriand, 87205 Saint-Junien, France; 7 Observatory of Institutional and Legal Changes (OMIJ), Faculty of Law and Economics, 5 rue Félix Éboué, B.P. 3127, 87031 Limoges, Cedex 1 France

**Keywords:** Advance directives, Qualitative research, Patient’s will, Ethics of care, End of life, Decision making

## Abstract

**Background:**

In France, advance directives are favourably perceived by most of the population, although the drafting rate is low. This ambivalence is challenging because advance directives are meant to promote the autonomy and freedom of choice of patients. The purpose of this study was to analyse the content of advance directives written by patients suffering from malignant haemopathies to better understand how patients put them into practice. These could be relevant as early as the initial diagnosis of haematological malignancies because of the uncertain course of the disease.

**Methods:**

This was a multicentre, qualitative, descriptive study. The advance directives written by patients with malignant haemopathies treated in one of the six French hospital departments were included in the study from 01/06/2008 to 15/04/2016. A thematic analysis of the advance directives was performed by two researchers: a senior haematologist and a research assistant.

**Results:**

The median age of the patients was 69. Most were women (sex ratio: 0.59), living as a couple (57%), with lymphoid pathologies (66%), who were still alive two years after the instructions were written (63%) and had nominated a health care proxy (88.6%). Free texts (62.9%) were richer in content than pre-defined forms. The advance directives were used in three ways: for a purely legal purpose, to focus on medical treatments or actions, or to communicate a message to the family. Three main themes emerged: (1) refusal of medical treatment (100%), in which patients express refusal of life-sustaining care (97.1%). The actual treatments or the moment when they should be limited or stopped were not always mentioned in detail. (2) A desire for effective pain relief to avoid suffering (57.1%) and (3) messages for their family (34.3%), such as funeral arrangements (17.1%) and messages of love or trust (14.3%).

**Conclusions:**

Patients who write advance directives are not necessarily at the end of their lives. Their content mainly conveys treatment wishes, although patients also use them to pass on personal messages to their close family. This emerging role of advance directives to communicate messages within the family should be valued, even if it is not their original purpose.

## Background

### Description of the French legislation on advance directives

It is first important to define the terminology used, as this research was conducted in France. The legal status of health care proxy (HCP) and advance directives (ADs) in France differ from that of other European or world countries. For this study, a HCP was defined, in accordance with French regulations [1], as a person chosen by the patient for two purposes: first, to support him/her in making decisions throughout the healthcare pathway and second to report the patient’s point of view and wishes concerning their end-of-life care, if they are unable to do so. The HCP is not a surrogate decision-maker because the burden of medical decisions remains with the physician in charge of the patient and not the relatives. The role of the HCP is strictly informative, and the final decision belongs to the physician. The law specifies that the opinion of the HCP prevails over any other family opinion, unless ADs have been drawn up by the patient. ADs are a written document by which the patient expresses his/her preferences in case he/she is unable to express them. The patient indicates his/her wishes concerning end of life care and the conditions for limiting or stopping treatment. In France, ADs were only given legal status in 2005 through law n°2005–370, related to patients’ rights and the end of life [2]. This law established, for the first time in France, an obligation for doctors to consider the wishes of the patient expressed in an AD. Initially ADs were strictly informative, and the final decision belonged to the physician. March et al. correctly states that the European attitude of a doctor’s liability, despite their patient’s wishes, is contrary to the American attitude, in which a patient autonomy is the main criterion for decision making [3]. The impact of culture on the acceptance of ADs in end-of-life decision making has been highlighted by several authors [4, 5]. In Latin societies, such as France, the previously expressed will of the person can be tempered by the intervention of professionals, close relatives, and the state, who/which can modify individual choice for the good of the person in the name of collective rule [5]. Aspects of modern end-of-life care should include patient involvement and the appropriateness of care. Towards this end, the medical culture needs the change that the law enables [6]. In daily practice the patient’s wishes are initially collected as administrative data upon hospital admission. Information booklets are often made available to patients without being integrated into the patient/medical team relationship [7]. Furthermore, the general public and health professionals are not sufficiently aware of the precise content of the law of April 22, 2005, explaining why few citizens write ADs [8]. It was only in 2016 that ADs acquired greater legal weight, but they are not binding [9]. Doctors must follow ADs except when: (1) a vital emergency occurs during the time required for complete assessment of the situation and/or (2) the content of the AD appears to be manifestly inappropriate or inconsistent with the patient’s medical condition. The decision to refuse to apply ADs is taken under the guise of three conditions: by medical committee, in consultation with an independent colleague and informing the HCP, or failing that, the family or close relatives. Ambiguity persists because, although the will of the person is reinforced by giving more weight to ADs, their restrictive force is still subject to the physician’s interpretation. ADs are currently perceived favourably by most of the population and are progressively integrated into the doctor-patient relationship, but without any impact on the percentage being drafted, as only 2.5% of patients have used them [10]. This ambivalence is challenging because ADs are meant to promote the autonomy and freedom of patients [7]. Reasons given are unfamiliarity with the concept [11], refusal to acknowledge their importance (83%) or anticipate or speak about the subject (22%), and lack of interest (36%) [12]. Given the poor uptake of ADs, it is understandable that very few studies concerning ADs that focus on patients with malignant haemopathies are available in the literature [13]. No publication has previously described the content of the ADs of patients with haematological malignancies. This is puzzling because this oncology specialty is one in which the evolution of the disease can be the most rapidly favourable or unfavourable. However, there are certain steps on the clinical pathway in which patients are confronted with important choices, such as receiving an allogenic bone marrow transplant, inclusion in early clinical research trials, or receiving solely palliative care. The patient is informed at each stage of his/her illness and cannot ignore the evolving risks. He/she decides with the haematologist which treatments he/she accepts and refuses. The purpose of this study was to analyse the content of ADs written by patients with malignant heamopathies to better understand how they put them into practice.

## Methods

This was a qualitative, descriptive, multicentre study carried out in two stages. Our first aim was to continue the preliminary work conducted in the Haematology and Cellular Therapy Department of the University Hospital (CHU) at Limoges, published in 2014: a mixed-methods study comprising retrospective analysis of a random sample of 200 patient medical records crossed with a qualitative analysis of the content of the ADs [14]. This first stage highlighted the factors associated with the designation of a HCP and writing of the ADs. Second, we prospectively collected ADs given directly by the patient to a healthcare professional, from six sites: The Haematology and Cellular Therapy Department of Limoges and five departments treating patients with malignant haemopathies [Hospitals at Libourne (33500), Saint Yrieix la Perche (87500), Saint Junien (87200), the Curie Institute (75005), and the Saint Antoine Hospital (75012)].

### Sample selection

ADs written by patients being treated for a malignant haemopathy in one of the six sites were included in the study from 01/06/2008 to 15/04/2016.

### Collection of the source data and ADs of patients at hospital departments included in the study

First, 200 medical records of patients presenting with a haematological malignancy and treated in the Haematological Department of the Limoges University Hospital from June 1, 2008 to April 30, 2012 were randomly drawn. Data were extracted by a research assistant using an abstraction protocol to perform a retrospective descriptive quantitative analysis. Study data included: whether a HCP had been designated, whether an AD had been written and was available, and the mention of a wish to meet a religious representative or a volunteer. Second, the qualitative study was carried out prospectively from May 15, 2012 to April 15, 2016, at six hospital sites that vary in the way they function and the type of patients under their care. Three were in urban areas: two public university hospitals, (Limoges and Saint Antoine (Paris)) dedicated to haematopoietic stem cell transplants and one private institute (Curie Institute), whereas two were in rural areas (< 100 inhabitants per km^2^, St Yrieix la Perche and Saint-Junien) and one was semi-rural (Libourne). They all offer the possibility for patients to indicate whether they have written ADs and designated a HCP during the hospital admission procedure. The Limoges CHU and Libourne Hospital both have this general administrative procedure and a proactive institutional procedure carried out by trained health professionals: all admitted patients are informed in person about ADs and HCP and those who are interested are provided with an AD (a blank sheet of paper in Limoges and a form in Libourne). The Limoges CHU offers the patient the possibility of a visit by a religious representative and/or volunteer to determine their desire to discuss religious and spiritual issues. Bordeaux proposes personal spiritual accompaniment.

Each healthcare professional from the six departments who met with a patient who had written an AD, handed it to the research assistant with the patient’s spoken agreement after information on the objective of the qualitative research had been explained to him/her. All sociodemographic and clinical data were anonymized and entered into an EXCEL worksheet by the research assistant for analysis.

### The Limoges University hospital department

The following data were extracted from the Medical Information and Evaluation Department (PMSI, Programme de Médicalisation des Systèmes d’Information) at Limoges CHU and only concerns patients admitted to the Haematology and Cellular Therapy Department from June 1, 2008 to April 15, 2016: sex, pathology, date of birth, marital status, date of diagnosis, and date of death. To analyse this data, a subgroup was created based on marital status: those living with a partner and those living alone (widowed, single, divorced, or separated). ADs were collected punctually by a health care professional. Since 2011, the Limoges Haematology Department has offered patients the possibility of meeting a research assistant trained in qualitative research, specialized in medical law, and qualified in clinical ethics. Patients wishing to discuss ADs may ask a health care professional to set up an appointment with the research assistant. Legal information is provided, but not editorial help, to patients wishing to write an AD.

### Other sites (*n* = 5)

We were unable to harvest the data for the patients with malignant haemopathies admitted during the study period at the other sites. We decided to describe the patient data we had in our possession for the Limoges site and focus on the content analysis of the ADs collected from the six sites. We included hospitals and structures of varying size and geographical location to describe the reality of ADs appropriation to not focus solely on a university hospital, for which the practices do not reflect the reality of those in everyday clinical services. We were informed of the ADs punctually collected by a health care professional in the five hospitals. We present an extended series of ADs from various sites with different haematological practices.

### Statistical analysis

Overall survival was calculated from the date of the ADs until death or last follow-up and are presented using the Kaplan-Meier method. Data concerning patient survival were updated on May 15, 2016.

### Qualitative analysis

The qualitative analysis of the ADs content was carried out using the same methodology as for the preliminary prospective study, consisting of the method described by Paillet & Mucchielli [15]. No specific qualitative data analysis software was used. Thematic analysis of the ADs content was carried out after having identified the principle messages and key words and determining their recurrence within the documents. This analysis was performed by a multidisciplinary research team composed of a senior haematologist and a research assistant trained in qualitative research, medical law, and clinical ethics. The anonymized ADs were first read by each of the two researchers to familiarize them with the data and then re-read several times to analyse each AD. The coding was defined, and the ADs coded by each of the two analysts, then compared, discussed, prioritized, and tested with the data before being assigned into related categories. There was a discussion of the themes and keywords that emerged from them at the time of the analysis. Any variance in interpretation was resolved by discussion among the researchers. Direct quotes are presented in italics bracketed by inverted commas. All patients whose ADs were analysed gave their oral consent that the content be shared once it had been rendered anonymous.

## Results

The first step of the descriptive study conducted retrospectively in the Limoges Haematology and Cellular Therapy Department resulted in the collection of six ADs in the medical charts. In the second step, 31 ADs were collected by the heath-care professional and given to the prospective research lead at the six hospitals sites. Overall, 37 ADs were collected, of which 24 were from the Haematology and Cellular Therapy Department of the Limoges CHU from an active list of 4423 patients from June 1, 2008 to April 15, 2016 (*n* = 24/4423; 0.5%). Thirteen ADs were collected from the other hospital sites: seven from Libourne Hospital, two of which were excluded as they had not been completed, (*n* = 5, ADs 22, 23, 25, 26, and 29); and six from the remaining four hospitals (Saint Antoine Hospital (*n* = 2, ADs 24 and 28), Curie Institute (*n* = 2, ADs 19 and 20), Saint-Junien Hospital (*n* = 1, AD 35), and Saint Yrieix la Perche Hospital (*n* = 1, AD 15). Finally, 35 ADs were retained for analysis. Patients wrote them over a period of 10 years, the oldest dating to April 1, 2006 and the most recent April 15, 2016. A substantially higher number of ADs were collected from the two sites which have a proactive institutional procedure carried out by trained health professionals – Limoges (*n* = 24) and Libourne (*n* = 5) – than those with a general administrative procedure (*n* = 6). The analysis of the ADs did not reveal a difference depending on the site where the patient was treated.

### Description of the patient sample

**Fig. 1:**
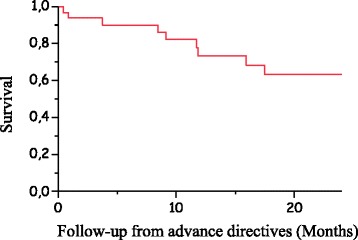
Survival curve

Patients who wrote ADs were more often female, with a sex ratio of 0.59 (22 women and 13 men), and had a median age of 69. More than half lived with their partners (*n* = 20/35, 57%) and two-thirds had lymphoid pathologies (*n* = 23/35, 66%). Table 1 presents the characteristics of the patient cohort. The haematological pathologies involved were severe, but the two-year overall survival rate was 63%. The survival curve is shown in Fig. 1. Among the 14 patients who died (*n* = 14/35, 40%), nearly all died either in the hospital or another institution, whereas only two (5.7%) died at home. Most of the patients who wrote ADs also nominated a HCP (*n* = 31/35, 88.6%): the spouse for 42.8% (*n* = 15/35); the next of kin for 28.5% (*n* = 10/35); or a sibling, friend, or doctor for 14.3% (*n* = 5/35). One person (2.8%) did not specify their relationship with the person chosen as the HCP. Table 2 describes the data of patients who nominated a HCP and their willingness to discuss end of life care. Indeed, two hospital sites offered either the possibility of meeting with a religious representative (Limoges) or proposed personal spiritual accompaniment (Libourne). At the Limoges CHU, 16.6% of the patients (*n* = 4/24) ticked the YES box, whereas, 100% (*n* = 5/5) at Libourne left it blank. Limoges also offered the possibility of being visited by a volunteer: 62.5% ticked the NO box (*n* = 15/24) and 33.3% left it blank (*n* = 8/24).

**Table 1:** Characteristics of the cohort of patients with malignant haemopathies

Centers participating in the study	CHU Limoges Haematology and Cellular Therapy Department: pts. admitted between 2008 and 2016 *n* = 4423 pts		Libourne Hospital Department treating pts. with malignant haemopathies	Other hospital Departments treating pts. with malignant haemopathies	Total ADs
Drafting of ADs	Yes *n* = 24	No *n* = 4399	*n* = 5	*n* = 6	*n* = 35
Demographic data					
Sex ratio	0.84	1.28	0.25	0.20	0.59
Median age [min-max] (years)	68[60–83]	69[16–99]	77[48–77]	75,5[63–85]	69[48–85]
Marital status (n;%)					
Patients living alone	8; 33.3	975; 22	2; 40	4; 66.7	14; 40
Married or with a partner	15; 62.5	2252; 51	3; 60	2; 33.3	20; 57
Not filled in	1; 4.2	1172; 27	0; 0	0; 0.0	1; 3
Format of the advance directives (n;%)					
Blank paper	17; 71	–	0; 0	4; 66.7	22; 62.9
Form	6; 25	–	4; 80	2; 33.3	11; 31.4
Dual	1; 4	–	1; 20	0; 0.0	2; 5.7
Group by years (n;%)					
2008–2011	8; 33.3	2800; 63.3	1; 20	0; 0.0	9; 25.7
2012–2016	16; 66.7	1599; 36.1	4; 80	6; 100	26; 74.3

*ADs* advance directives, *Pts* patients, *CHU* University Hospital, Other hospitals (Curie Institute, Saint-Antoine Hospital, Saint Yrieix la Perche Hospital, Saint-Junien Hospital); Patient living alone (widowed, single, divorced, separated)

**Table 2:** Description of patients who nominated a heath care proxy and their willingness to exchange on end of life issues

Centers participating in the study	CHU Limoges Haematology and cellular therapy Department *n* = 24			Libourne hospital Departement treating pts. with malignant haemopathies *n* = 5			Other hospital^a^ Departments treating pts. with malignant haemopathies *n* = 6			Total ADs *n* = 35
ADs written	Yes	No	NS/NA	Yes	No	NS/NA	Yes	No	NS/NA	
Designation and identity of the heath care proxy (n;%)										
HCP designation	22; 91.7	0; 0	2; 8.3	5; 100	0; 0	0; 0	4; 66.7	2; 33.3	0; 0	31; 88.6
Spouse/Partner	13; 54.2	0; 0	0; 0	1; 20	0; 0	0; 0	1; 16.7	0; 0	0; 0	15; 42.8
Next of kin	5; 20.8	0; 0	0; 0	4; 80	0; 0	0; 0	1; 16.7	0; 0	0; 0	10; 28.5
Other^a^	3; 12.5	0; 0	0; 0	0; 0	0; 0	0; 0	2; 33.3	0; 0	0; 0	5; 14.3
Unspecified	1; 4.2	0; 0	0; 0	0; 0	0; 0	0; 0	0; 0	0; 0	0; 0	1; 2.8
Wish to meet a volunteer (n;%)										
	1; 4.2	15; 62.5	8; 33.3	–	–	–	–	–	–	1; 2.8
Wish to meet a religious representative (n;%)										
	4; 16.6	13; 54.2	7; 29.1	–	–	–	–	–	–	4; 11.4

^a^*ADs* advance directives, *pts.* patients, *NS* not specified, *NA* not applicable, *CHU* University Hospital, Other hospitals = Curie Institute, Saint-Antoine Hospital, Saint Yrieix la Perche Hospital, Saint-Junien Hospital, *HCP* health care proxy, Other: sibling, friend, doctor

### Format of the ADs

Patients either expressed themselves freely, writing on a blank sheet of paper, or filled in a printed form. Most patients wrote their ADs on blank paper (*n* = 22/35, 62.9%). Eleven chose to fill in a form (*n* = 11/35, 31.4%): seven of which were provided by the institutions (four from a hospital (ADs 16, 22, 27, and 35), three from the French Health Insurance (ADs 15, 18, and 30), and four from a pro-euthanasia association (ADs 9, 11, 12, and 32). Standard ADs forms from national organisations, such as the *Haute Autorité de Santé* (HAS – equivalent to NICE in the UK), are composed of predefined topics, with a list of questions concerning treatment, or a list of treatments, to which the patient answers YES, NO, or I DON’T KNOW [16]. Most patients merely ticked YES or NO or indicated acceptable or refused treatments, with very few taking advantage of blank spaces. Two patients completed a form as well as detailing their wishes on a blank sheet of paper (*n* = 2/35, 5.7%, ADs 29 and 31).

### Analysis of the ADs content

Qualitative analysis of the 35 ADs identified the jargon used by some patients to name the text: *“Declaration of my advance directives”* (AD 7), *“Life end dispositions”* (AD 3); *“Will”* (AD 24). Some used possessive adjectives: *“Wishes concerning the end of my life”* (AD 35), *“My last will and testament”* (AD 33). Others did not name their text, but addressed the ADs to their healthcare professionals or the hospital: *“To the doctors...”* (ADs 1 & 5), *“To the care personnel…”* (AD 8), *“To the Limoges CHU...”* (AD 10).

### I. Legal approach to the concept of ADs

Most of the patients dated and signed the ADs and specified their identity and date and place of birth as legally required (*n* = 24/35, 68.6%). The patients sometimes used legal wording: “*I the undersigned”* (AD 30); “*For all due intents and purposes*” (AD 3). The articles from the French Public Health Code relating to refusal of life-sustaining care or the treatment of pain were sometimes quoted by patients verbatim. Some patients quoted the law which stipulates that “*Procedures that appear ineffective or disproportionate, or which have no other effect than the artificial sustaining of life, may be suspended or not performed*.” (ADs 14, 28, 9, 11, 12, 29, 31, and 32). However, no patient used the terms “unreasonable persistence” or “theory of double effect” which are part of the legal terminology used since 2005, possibly due to a general lack of awareness of these terms.

Patients referred to their mental capacity at the time of drafting (*n* = 26/35, 74.3%) and also to writing without constraint (*n* = 10/35, 28.6%) as a way of validating what they had written: *“Being in full possession of my mental faculties”* (AD 25), *“Being of sound mind”* (AD 21), *“I, the undersigned, enjoying full capacity of my civil rights and mental faculties, acting in full awareness and complete freedom ...”* (AD 7), *“Declare that I am writing this document in complete freedom, with no external pressure and in full possession of my faculties.”* (AD 17).

The patients continued their writing, envisaging the possibility that they would not be in a position to express their wishes in the future, either quoting the legal formula or using equivalent expressions: *“If I should find myself incapable of expressing my wishes following a serious accident or an incurable illness that affects my physical integrity and my mental faculties”* (AD 7*); “If I find myself unable to express my wishes”* (ADs 13 & 32); *“If I am myself incapacitated verbally, physically, or psychologically from expressing them or if my mental faculties are reduced …”* (AD 33).

The place where the ADs were to be kept was detailed in the forms provided by the pro-euthanasia association (ADs 9, 11, 12, 29, 31, and 32) and by three patients using plain paper (ADs 3, 4, and 17).

Apart from a legal approach to writing the texts, the patients also wrote ADs in two other ways: either drafting them according to legal stipulations, focusing on the medical treatment, or by adopting a more personal approach.

### II. Description of medical treatments or actions

Some patients described their wishes in less formal language and indicated overall directives for their treatment: *“I would be grateful if you would be kind enough to take my recommendations into account”* (AD 1); *“These are my directives…”* (AD 2); *“I thank you in anticipation of the attention you will pay me.”* (AD 4). The way they expressed themselves varied: the patient either expressed what he or she wanted, by demanding that his/her wishes be respected, or with a more neutral formulation expressing general concerns about their treatment to the medical team: *“I, firmly desire, in all lucidity, serenity and certitude, the application of the following end-of-life procedure... All my wishes are irrevocable, maturely and fully thought out.”* (AD 35), *“I entreat you that I be spared any excessive or futile therapy.”* (AD 3).

The subjects covered by the ADs were common to any advanced phase of a serious illness and yet, specific concerning the treatment of a haematological disorder. In decreasing order of frequency, patients refused excessive or futile therapy (*n* = 34/35, 97.1%), requested effective pain management (*n* = 20/35, 57.1%), or even requested sedation (*n* = 8/35, 22.8%). A few patients referred to euthanasia (*n* = 6/35, 17.1%) or suicide or assisted suicide (*n* = 3/35, 8.6%). The five forms from a pro-euthanasia association refer to it as follows: *“If there is no hope of returning to a conscious and independent life, that they should ensure a rapid, gentle death.”* (ADs 9, 11, 12, 29, 31, and 32). Three patients mentioned suicide and assisted suicide: *“Perhaps I will put an end to my days if changes in my state [of health] suddenly reduce my freedom of will.”* (AD 5), *“Struck by an incurable neurological disease, I have decided to commit suicide.”* (AD 29*), “If a law allowing assisted suicide is passed, I desire to be offered this solution.”* (AD 17).

### Theme 1: Refusal of excessive and futile therapy

In terms of limiting or stopping treatment, few patients managed to clearly articulate what treatment options were important for them. Most patients were happy to indicate: “*I refuse any excessive or futile treatment.*” (ADs 4 and 16), “*I do not wish for any excessive or futile treatment.*” (ADs 8 and 10), “*I ask that all the treatments that keep me artificially alive are stopped.”* (AD 20), “*I do not want any excessive or futile treatment.*” (ADs 13 and 25), without further defining what they considered to be their treatment limits. Only a few patients described what they meant by excessive or futile therapy in concrete terms: the refusal of specific treatments, such as transfusions, a new chemotherapy line, artificial feeding, disproportionate additional tests, transfer to the Intensive Care Unit, or invasive procedures or surgery: *“I absolutely refuse any attempt at resuscitation.”* (AD 4), *“No chemotherapy, no radiotherapy, no surgery may be carried out without my express agreement.”* (AD 17), *“I refuse COMPLETE BLOOD TRANSFUSIONS, red blood cells, white blood cells, platelets, or plasma under any circumstances.”* (AD 22), *“No to artificial feeding.”* (AD 23), *“I do not want chemotherapy for the treatment of my myeloma. Nor do I wish to be resuscitated.”* (AD 35), *“I, resolutely and in all lucidity, serenity and certainty, want the application of the following medical end-of-life process: I refuse any treatment by chemotherapy, inserted...”* (AD 33).

Two people described why they refused certain treatments. A woman asked to make quality of life the priority at the end of her life*: “What I like more than anything else is to be in my garden; I haven’t been able to garden for so long... I am exhausted, I do not want any more treatment. I have told my family that I am approaching the end of my story.”* (AD 34). A man described a diminished quality of life: *“A very great and increasing structural weakness drives me from my armchair to my bed where I drowse or sleep... I ask that my life is not prolonged ... I have been kept alive by transfusions for more than four years...”* (AD 5).

Refusal of technology was also highlighted, whether it be for investigation – *“I do not want examinations such as IMR scans, PET scans, CT scans, scintigraphy, X-rays, checks etc...”* (AD 33) – or invasive techniques – *“I refuse to be attached to a machine to be kept alive artificially or any other disproportionate treatment. I ask that any life-support machine keeping me alive be turned off.”* (AD 33), *“I insist on being spared any artificial life support.”* (AD 3), *“I refuse any artificial feeding through a tube. I refuse any artificial ventilation, with the exception of simple oxygen therapy.”* (AD 17).

Only two people mentioned the illness they were suffering from: one patient referred to his personal experience of the illness and that of his family to explain his fear of suffocation, which he did not want to experience again: *“At the age of 6, when I had the C... illness... I suffocated... I do not want ever to relive that ... My own father died of suffocation at home ... no longer able to express himself ... unconscious.”* (AD 5); *“I do not want chemotherapy for the treatment of my lymphoma.”* (AD 35). One person showed a very clear understanding of the nature of his cancer: *“I know I have a cancer for which there is no cure. I have been informed of its foreseeable development. I have understood that I am likely to be rendered incapable of knowing my wishes. I have also understood that I am likely to have respiratory and nutritional problems not compatible with life.”* (AD 17). Others referred to the illness they were suffering from without naming it: *“My illness.”* (AD 16).

While treatment limits are a recurrent theme, only a few patients indicated at what point in time the treatments should be limited or terminated. Those who did, highlight the importance of three key points: maintaining their functional and mental independence, their capacity to communicate with other people, and making decisions for themselves.

### Loss of functional or mental independence as a criterion for limiting or terminating treatments

The patients underlined the importance of their functional independence and their ability to express themselves, which sometimes led to the refusal of treatment that may have the effect of reducing their mobility or their state of consciousness: *“No to taking any medicine that could cause the loss of residual faculties (sight, mobility, bodily functions).”* (AD 2), *“I wish to be hospitalised when I am unable to carry out everyday life activities unaided.”* (AD 34)*, “I do not want other treatments, what I like is gardening, with the treatments I am exhausted.”* (AD 13), *“Within reason. I do not want to be a “vegetable” for several years*.*”* (AD 23). The form from a pro-euthanasia association refers to mental and physical independence as being criteria for the continuing or terminating treatments: *“If there is no hope of a return to a conscious and independent life, let me be provided with a gentle death in as much as I find myself in a situation where my physical or neuro-psychic faculties are deteriorating without hope of improvement.”* (ADs 9, 11, 12, 29, 31, and 32).

### The ability to communicate with others

The importance of communicating and being able to speak with others was highlighted by the patients: “*In any situation where I am not able to communicate clearly, contact my husband* (whom the patient had designated as HCP) *before any measure mentioned above is taken, or indeed any measure having significant potential side effects.”* (AD 23), *“Yes to the absorption of sedatives to avoid pain preventing me from expressing myself serenely.”* (AD 2), *“I refuse any excessive or futile therapy which would leave me deprived of my mental faculties.”* (AD 3), *“If I found myself in a situation where my neuro-psychic faculties were deteriorating without hope of improvement…”* (AD 7). Being able to decide for oneself and not be dependent on others was mentioned in certain ADs: *“If changes in my state of health suddenly reduced my freedom of will.”* (AD 5).

### Theme 2: Effective pain treatment

More than half of the patients expressed a desire for effective pain relief to avoid suffering (*n* = 20/35, 57.1%). Some mentioned their acceptance of the risks associated with pain relief treatments that may shorten their life (*n* = 16/35, 45.7%). The patients expressed their wishes concerning the treatment of pain and symptoms as follows: *“Above all, I wish my pain to be relieved effectively, even if a side effect of that could be to shorten my life.”* (AD 14); *“I wish my pain to be relieved effectively, even if a side effect of that could be to shorten my life.”* (ADs 17 and 20), *“That, above all, the treatments serve to lessen the pain.”* (AD 25), *“I also ask the medical teams to do all they can to relieve my physical and psychological suffering effectively, even if that has the effect of shortening my life.”* (AD 28), *“I ask that I be administered appropriate medicines to effectively relieve the pain caused by the illness (or the cessation of life-sustaining treatments), even in circumstances where it could shorten my life.”* (AD 7*).* Three people referred to sedation: *“Pharmacological sedation should be implemented as soon as my comfort can no longer be assured.”* (AD 17)*, “Yes to sedation, in extreme circumstances only.”* (AD 23), *“I accept to be plunged into total unconsciousness. I ask for an artificial coma.”* (AD 33) and five on the standard forms (ADs 22, 23, 26, 31, and 32). Only two patients referred to palliative care (ADs 2 and 10).

### III. A personal interpretation of the concept of ADs (*n* = 12/35, 34.3%)

Some of the patients used their ADs to pass messages to the care team (mentioning family disagreements or people to contact if their clinical state deteriorated) or to report who should be informed within the family: *“The person to advise in case of my death is my husband. I wish you to know I have great reservations about information being given to my daughter.”* (AD 1), *“People who are only to be informed when the terminal phase begins: (...)”* (AD 4), *“This decision was made after having told my husband and my son.”* (AD 21), “*Original given to Doctor X, a copy to each of my three children.*” (AD 3).

In the same way, some expressed their trust in their care-givers*: “I do not want any futile treatment if the doctors think it is unnecessary.”* (AD 13), “*I ask firmly of the doctors who have treated me during my illness, and whom I thank for having cared for me, not to proceed with any futile treatment on my behalf at my life’s end.”* (AD 24).

Some patients used words such as “*decease”* (AD 1) and “*death*” (AD 2), and terms used in the forms produced by the associations included *“Gentle death... the right to die with dignity”* (ADs 9, 11, 12, 29, 31, and 32), *“If I were to die,”* (AD 8). On the contrary, some used euphemisms to speak of their death: *“Finish my life’s journey”* (AD 4), *“Leave for the yonder”* (AD 10), *“May they help me depart”* (AD 27), but the patients rarely used the expression “end of life”. (ADs 3 & 33). At the same time, references were made to life in different ways: *“Life is a wonder”* (AD 5), or patients indirectly described the end of life: *“For me, to prolong my life at any cost is not a priority. I refuse certain treatments the sole purpose of which is to keep me alive.”* (AD 25), *“I certify that I refuse any care intending to prolong my life.”* (AD 21), or *“artificially prolonging my life”* (ADs 17 & 28), sometimes quoting the law *“side effect of shortening my life”* (ADs 7, 9, 11, 12, 14, 15, 17, 18, 20, 22, 23, 28, 29, and 30–32).

The ADs also disclosed intimate feelings of the patients, expressing introspection*: “I am keen to be present at my death to the extent that this is possible.”* (AD 2), *“My deeply held wish is to finish my life’s journey with the best support conditions so that it takes place in all serenity.”* (AD 4), *“My life is not to be prolonged, I have had my time, I have been kept alive by transfusions for more than 4 years. Life is a wonder, but even the best things must come to an end. I will have lived enough...”* (AD 5), *“I cannot cope with any more new treatments, I don’t see the point...”* (AD 13). Respect and personal dignity were also brought up: “*Claim the right to die with dignity.”* (AD 10), *“I sign these ADs after long reflection. They are the expression of my last free wishes. I want them to be honoured.”* (AD 17), *“For me, to prolong my life at any cost is not a priority. I refuse certain treatments the sole purpose of which is to keep me alive. When my illness becomes too serious, I do not want any futile treatment. Do what is within your power to allow me die with dignity and due personal respect.”* (AD 25), *“What I am asking is to die with dignity.”* (AD 33).

### Theme 3: Personal messages for their family

Some ADs referred to funeral arrangements (*n* = 6/35, 17.1%) or to where the patient wished to die: “*I would like to inform you that I do not wish to die in L..., I wish to be taken back to the C... hospital, closer to where I live.*” (AD 1), “*If I were to die, my wish is to be cremated*.” (AD 7), “*After my death I wish to be cremated and my ashes to be deposited in the family vault.*” (AD 11), “*I hereby express my wishes for the organisation of my funeral: to remain in the hospital morgue... the ceremony to be most strictly intimate*...” (AD 13), “*I wish to be cremated in a pine coffin, I wish to have a blessing in the church surrounded by my close friends and relations. I wish my ashes to be scattered in the commemoration garden near my parents*.” (AD 19), “*I wish to be cremated. For the ceremony contact…”*(AD 22).

Four patients stated their being in favour of or against organ donation: “*I am in favour of organ donation*” (ADs 9 and 26), “*I wish my body to be kept intact”, “I do not wish to donate my organs and tissues*.” (ADs 8 and 22).

Some of the writing contained an emotional dimension with the ADs being used as a way to transmit messages of love and trust to friends and family (*n* = 5/35; 14.3%): “*Should my faculties deteriorate, I have every confidence that my wife or one or other of my six children will keep to the spirit of my directives*.” (AD 2), “*In any situation where I am not able to communicate clearly, contact my husband* (whom the patient had designated as HCP) *before any measure mentioned above is taken, or indeed any measure having significant potential side effects.”* (AD 23). One patient chose flowery stationary to personalize her ADs and to thank her family: “*To my family which has supported me throughout all these difficult years... I love you all with all my heart and thank you for these years of happiness spent with you*.” (AD 24), “*I have spoken of it to my husband, my children and my grandchildren*.” (AD 34).

## Discussion

The present qualitative analysis of the content of ADs written by patients with malignant haemopathies is the first of its kind and provides insight into how these patients perceive the process. ADs, together with the nomination of a HCP, legally empowers a patient to have his/her wishes respected, reinforces their independence within the health system and, more broadly, within general society. During recent years, an increasing number of patients covered by the French health system are nominating a HCP to speak on their behalf in anticipation of a time when they are no longer able to express themselves: 31% for cancer patients [17], 40.9% for dependent elderly people [17], and up to 64.5% for patients in a haematology department [14]. However, only a small percentage of patients write ADs in France: from 1.5 to 6% [12, 18–20]. Patients in oncology and haematology departments typically have more severe disease, sometimes at an advanced stage, and are undergoing treatments, such as bone marrow allografts, which may be fatal. ADs are thus even more important for this population. Our study, conducted in such a setting, further highlights the small number of patients actually writing ADs, in spite of a law designed to help the patient indicate his or her wishes before potentially becoming incapacitated. One of the most significant barriers to using this legal tool is the subject itself: anticipating one’s incapacity or mortality is frightening [21].

Concerning the HCP, the stakes are different, because the person normally makes choices in a family environment, whereas the ADs position the person in the context of therapeutic limitation, which is difficult to define and sometimes involves unknown techniques. Our detailed presentation of ADs content contrasts with that of other studies conducted in oncology and palliative care, which present the broad themes expressed by patients in ADs [22, 23].

### Patient profiles

Most of the ADs were written by older women (median age: 69), as previously reported in single-centre studies [24, 25]. This also reflects the findings of a 2015 Belgian study of the general population showing that women were 1.5 times more inclined to talk to their doctor about their wishes concerning medical treatment than men [26]. The Mayo Clinic found a similar median age at the time of writing an AD of 67 years in a study carried out in 2007 [19] on the profile and preferences of cancer patients who draft ADs [22]. Most of the ADs in our sample were written by patients living as a couple who frequently designated a HCP, often their spouse. This finding is also concordant with the literature [11, 27]. Thus, an elderly woman living in a couple, is more of a predictor of drafting an AD than a patient seeking a true spiritual quest, given the very small proportion of patients wishing to be supported by a religious representative [17]. Our results also show that the trigger to draft ADs is not just the fact that patients are approaching the end of their lives, as more than two-thirds of our patients were still alive two years after they had written their ADs. As conventionally described in the literature [28], the patients of our sample who wrote ADs had three types of profiles: autonomous, intermediate, and paternalistic. Those with an autonomous profile were well-informed, used legal terminology, and were aware of the implications of their decision to limit or terminate one or more treatments (*n* = 24). They demanded that their wishes be respected: some insisted on deciding for themselves and others claimed their rights to choose or refuse treatments. They show evidence in their writings of personal or familial experience of end-of-life, expressing their refusal to relive it, as in the case of a patient who had been deeply marked by the death of his father and asked for optimum pain relief. Others expressed their philosophy of life, which enabled/allowed them to accept the uncertainty of their prognosis, with death as a possible outcome. Most details of the treatments they accepted or refused, as well as the precise moment when the treatments were to be stopped was sometimes not made very clear. Criteria for limiting or stopping treatment varied from one person to the next, with loss of independence, the ability to communicate, the ability to decide for themselves, and the quality of life, as well as the refusal of technology being cited. Some patients had approached a pro-euthanasia association to affirm their refusal of futile care and their wish for effective pain relief and terminal sedation, should they be in an incurable state and in unbearable pain, as well as a rapid and gentle death if there no longer existed any hope of return to a life of consciousness. Patients with an intermediate profile preferred a joint decision along the lines of “*I am giving lines of general guidance, but I trust my doctor to make medical decisions*” (*n* = 10). Finally, patients with a paternalistic profile referred to the confidence they have in their doctor and left it to them to decide when the time has come (*n* = 1).

### Type of ADs: Blank paper or standardized form

Nearly 70% of our patients preferred to write on plain paper. The psychological impact of filling in a form or freely expressing oneself on plain paper is different. It is easier to fill in a form, but the implications with respect to the end of life are more limited, which means the person is not encouraged to truly reflect on the matter [7]. Our results are concordant with those of a 2014 Swiss study which reported a lack of individualisation of texts when standardised forms are used, and the need to offer blank spaces to encourage patients to go into detail about their preferences concerning treatment in cases of critical situations or the end of life [29]. Those who chose to write their ADs on plain paper sometimes also gave an emotional dimension to what they wrote, which is absent from forms, the main purpose of which is to avoid technological treatments at the end of life. Recent ADs models proposed by French legislation give examples of treatments and invite the patient to state whether he or she accepts or refuses them, and to personalise the text using blank spaces. The patient may detail: “*Information or requests that I wish to express apart from my ADs.*” such as personal information, fears, expectations, and convictions. It is nevertheless stipulated that only information of a medical nature will constitute directives that doctors are obliged to observe [30].

### The ways in which patients use ADs

The size of the hospitals and their geographic location had no influence on the way patients wrote their ADs. The patients in our sample used the ADs in three ways: (1) to mainly focus on medical information with a more or less detailed description of the treatments they are or are not prepared to accept, (2) in a purely legal context, or (3) to add a personal dimension to what they wrote.

Most of the texts focused on medical information. Although no patient expressed the wish to undergo all possible treatments, in accordance with the low percentage (1.9%) reported by an American study conducted in 2010 [31], a third described which treatment they desired. When the patient declared refusal of futile therapy a generic formulation was often used – “*I do not want any futile treatment*” – without defining what this actually meant to them. Few of the texts were sufficiently detailed to be of practical use, leaving it to the care teams to define futile treatment.

More than half of the texts displayed a legal approach to the concept of the ADs, using legal terminology, sometimes quoting in extenso articles of the law. This suggests that these patients wanted their ADs to be taken seriously. This could stem from worries about credibility, the fear of making a mistake in formulating what they want, or possibly fear of a medical-legal challenge. It is not easy to compare our results with others, as most other studies only covered the main themes broached in ADs (place of death, the values of the person, the context in which the ADs are to apply, measures requested or refused, the HCP) rather than the content [29]. A recent French publication suggests that we are too keen to take refuge behind legal standardisation and that is unrealistic to expect a law to legislate the complexity of specific end-of-life situations [32]. We believe that healthcare teams should prioritise ethical over legal considerations, so as to make decisions that are the most closely matched to the patient’s wishes and interests as possible [21, 28].

It is in this spirit that some patients personalized their ADs. The ADs are not solely seen as a tool to convey information relating to their medical treatment, but also as a way of passing on personal information that they have been unable to talk about to those dear to them: they speak of confidence, love, or funeral arrangements. This use of ADs is not the uppermost purpose of the legislators and appears to reflect society’s shortcomings when it comes to talking about mortality and the end of life. Indeed, it is the essence of the care and relationships with others that this type of communication favours, over and above information of a practical nature for the medical team. However, health professionals want practical information giving them a clear idea of the patients’ wishes concerning whether the continuation of their care is unreasonable or limitation or termination of the treatment is desirable when they are unable to express them themselves. Thus, legislators, along with a section of the medical team, ask that ADs be as precise as possible to ensure the most effective treatment and care. This raises the question of whether standardised forms covering various diseases and treatments are preferable to forestall this imprecision? We would argue that standardising the doctor-patient relationship in this way fails to consider the patient’s vulnerability. This also raises the question of the pertinence of ADs models which detail the various treatments drawn up by scientific societies in the light of what the patients write in their ADs [33]. Furthermore, the more binding nature of the ADs should have an impact on the doctor-patient relationship and the decision-making process. However the mentality of the medical community must evolve, because some physicians indicate that ADs have no influence on their decisions [34]. This dichotomy between ADs and medical care has been demonstrated by several studies [35–38]. The binding nature of ADs should be explained to doctors because it changes the decision-making process. Writing ADs would be made easier if more importance was placed on the values of the person rather than factual descriptions. Patients sometimes find it hard to describe clinical and therapeutic situations. Therefore, a patient may find it easier to express his or her care and treatment expectations by means of a standardized form. The low number of ADs written prompted Zeisser and Weber to propose the development of ADs from a general historical notion to a more practical concept, including the values of the person [21]. The ADs drafting process must be genuinely integrated into a progressive system of early discussion [39, 40]. Advance care planning is a good way to integrate the patient’s wishes to pass on information to his/her family, because it is an ongoing process in which patients, families, and healthcare providers discuss the patient’s own values and preferences [41]. They discuss how they could guide current and future medical care and ultimately use this information to accurately document future health care choices. Advance care planning is a way to develop communication between all actors involved, which can drive specific medical treatment decisions, although it is not the primary intent of discussions.

### Future developments

The recent French law to further defend patient’s rights at the end of life includes measures to involve the general practitioner in supporting patients to draft ADs [1]. This was underlined by a recent German study of patients in palliative care, good health, or suffering from a chronic illness [29]. An alternative is to involve someone who is not part of the care team, representing civil society, and whose competences in terms of ADs could assist both the care teams and patients who request support. This option was chosen by the Limoges site as early as 2011, before the new law was passed. Each patient requesting information about ADs meets a research assistant who is familiar with their case and specialised in law and ethics. Thus, patients’ wishes concerning not only treatment options, but also how they envisage their end of life, can be better taken into account.

We believe that standardising ADs into a form to be filled in by the patient on admission reduces the ADs to just another administrative procedure rather than encouraging the patient to embark on personal reflection. It would not respond to the societal challenges inherent to the end of life and deny patients the opportunity to express their subjectivity, choices, and desires which can only belong to them [33].

### Study limitations

The main limitation of the study is that it was conducted in different-sized hospitals. Moreover, it may be informative to compare the content of ADs written in France with those written in the US or continents with different cultures. Inclusion of only patients with a haematological malignancy may not have allowed us to assess the needs of patients suffering from less serious diseases.

## Conclusion

In France, few patients with malignant haemopathies write ADs, as this can be perceived as a complex and anxiogenic process. As members of care teams involved in accompanying patients at the end of their lives, we believe that more can be done to support patients in writing their ADs. Our results highlight that ADs are not limited to end-of-life patients, and that patients use them to pass on personal messages to their loved ones, in addition to expressing their wishes concerning treatment choices. This emerging role of ADs encourages intrafamilial communication and should be valued, even if it is not their primary purpose. People in the general population should be made more aware of the concept of ADs so that they can already think about it before the onset of disease and discuss these issues with their loved ones outside of an emotional context. Each patient should be given the possibility of writing their own ADs, an exercise that requires personal reflection. Nevertheless, the writing and legal formalization of ADs should consider the dialogue between the patient and his/her doctor, relatives, and caregivers, and respect the patient’s choices.

## Abbreviations

AD: Advance directives
CH: Local hospital
CHU: University Hospital
HCP: Health care proxy
